# Emergency department attendance for injury and behaviours suggestive of attention deficit hyperactivity disorder (ADHD): a cross-sectional study

**DOI:** 10.1186/s12887-020-02166-x

**Published:** 2020-05-29

**Authors:** Ester Conversano, Alice Tassinari, Lorenzo Monasta, Aldo Skabar, Matteo Pavan, Alessandra Maestro, Egidio Barbi, Giorgio Cozzi

**Affiliations:** 1 Department of Medicine, Surgery and Health Sciences, University of Trieste, Strada di Fiume 447, 34149 Trieste, Italy; 2 Clinical Epidemiology and Public Health Research Unit, Institute for Maternal and Child Health - IRCCS “Burlo Garofolo”, Trieste, Italy; 3 Department of Pediatrics, Institute for Maternal and Child Health - IRCCS “Burlo Garofolo”, Trieste, Italy; 4 Pharmacy and Clinical Pharmacology Unit, Institute for Maternal and Child Health-IRCC “Burlo Garofolo”, Trieste, Italy

**Keywords:** Screening, Treatment, Neurodevelopmental disorders, Inattentiveness, Impulsiveness, Trauma

## Abstract

**Background:**

The study aimed to investigate if the behaviours suggestive of ADHD were more frequent in a population of children attending the Emergency Department (ED) for injuries, rather than for other causes.

**Methods:**

A cross-sectional study was carried out. Patients, aged 6 to 17 years, attending the ED for acute injuries and other causes were considered cases and controls, respectively. We used a questionnaire, which investigates the presence in the child of inattention, hyperactivity, and impulsivity. The primary outcome was the number of children with behaviours suggestive of ADHD in cases and controls.

**Results:**

Five hundred forty-five children were enrolled, 251 with injuries and 294 with other complains. Twenty two out of two hundred fifty one (9%) children visited for injuries, and 30 out of 294 (10%) visited for other causes had behaviours suggestive of ADHD (*p* = 0.661). Among these cases, children with evocative ADHD scores had a higher probability (OR 4.52; 95% CI 1.45–14.04; *p* = 0.009) of having had more than five previous ED accesses due to injury, compared to the others.

**Conclusions:**

This study did non shown a difference in behaviours suggestive of ADHD between cases and controls, but identified a population of children with behaviours suggestive of ADHD who more frequently access the ED for injuries.

## What is already known?

- During their life, patients with ADHD are at risk of repeated injuries.
- The number of injuries could be decreased by an early diagnosis and appropriate treatment, but there is no agreement about a screening for ADHD in children accessing the ED for injuries

## What is new?

- There is a population of children with behaviours suggestive of ADHD with a history of repeated ED accesses for injuries.
- Addressing this population of children, the development of a specific screening tool for behaviours suggestive of ADHD could be considered.

## Background

Attention-deficit/hyperactivity disorder (ADHD) is the most common neurodevelopmental disorder in children and adolescents, with a prevalence that varies between 3 and 5% [1]. It is characterised by inattention, hyperactivity and impulsivity, causing impairment of daily activities [2–5].

Accidents in children and adolescents are the most common cause of visits to the emergency department (ED) and among the leading causes of morbidity and mortality in Europe [3]. Previous studies suggest that children affected by ADHD are exposed to a higher risk of severe accidental injuries due to hyperactivity and decreased vigilance [6–13]. Moreover, evidence shows that early diagnosis and treatment significantly reduces ADHD related comorbidity [14, 15]. However, no current indication exists to identify the presence of ADHD in patients who are admitted to EDs for repeated or severe traumas.

Previous studies performed in a paediatric ED setting did not reach a decisive verdict regarding any increased risk of injuries in children affected by ADHD [16, 17]. This study aimed to assess whether the frequency of visits to our paediatric ED due to trauma or injury was higher than the frequency of those who accessed the service for other causes among subjects with inattention and hyperactivity behaviours. The primary study outcome was to determine the number of patients with behaviours suggestive of ADHD in cases and controls. The second was to compare the rate of past injuries reported by parents among children with positive and negative scores for ADHD behaviours in all the cases examined in our ED.

## Methods

A cross-sectional study was carried out from May to September 2017 at the paediatric ED of the tertiary care children’s hospital Institute for Maternal and Child Health – IRCCS “Burlo Garofolo” of Trieste, Italy. The study protocol received approval from the Bioethics Committee of Friuli Venezia Giulia (CEUR-2017-Os-124-BURLO).

Patients eligible for the study were children attending the ED aged from 6 to 17 years old. The exclusion criteria were non-Italian speakers, patients with reported developmental delay, irreparable hearing, visual or intellectual delay, musculoskeletal or neurological diseases and non-self-caused-injury. Enrolment was carried out for approximately 6 hours per day, in the presence of a specially trained research assistant.

Children attending the ED for acute injuries, defined as trauma, wounds or burns, were included in the study group. Children attending the ED for causes other than injuries were included in the control group.

After the usual ED care and before the hospital discharge, the research assistant approached the parents of the injured and non-injured children for the enrolment. All children’s parents signed informed consent to participate in this study. We collected data on age, sex and nursing triage category of each patient using the Italian national triage category system consisting of four priority levels with increasing severity [from white (not urgent), to green (minor urgencies), yellow (urgent) and red (emerging/resuscitation)]. Parents were asked to complete the SCOD-Parent Rating Scales-Revised questionnaire for ADHD symptoms [17]. Parents were also asked how many times their child had visited the ED or been admitted to hospital for treatment of trauma, fractures and/or wounds needing suturing previously, and if the child was previously diagnosed with ADHD or was treated for ADHD.

The SCOD-Parent Rating Scale is a revision of the Disruptive Behaviour Disorder Rating Scale translated and adapted into Italian, and extensively tested and implemented in the Italian population. The SCOD-Parent Rating Scale questionnaire consists of 42 items following the criteria of the Attention Deficit Hyperactivity Disorder of the Diagnostic and Statistical Manual of Mental Disorders. The answers to each question are based on a rating scale from “0 = never”, “1 = occasionally”, “2 = often”, or “3 = very often” [17, 18]. As the aim of the study was not to diagnose ADHD in an ED setting, but rather to assess the presence of inattention and impulsivity as behaviours suggestive of ADHD, we requested answers to only nine of the SCOD questions related to these two symptoms. The behaviours suggestive of ADHD were defined by a score higher than the threshold of the inattention (≥14 points, range 0–27) and impulsivity / hyperactivity (≥12 points, range 0–27). These cut-offs represent the fifth percentile for these symptoms for males. Females have lower cut-off scores, 11 and 9 respectively, but we decided to use the cut-off for males for the entire population, in order to include only females with highly suggestive symptoms.

### Statistical analysis

Data were described as frequencies and percentages, and as medians and interquartile ranges (IQR). Statistical significance (*p* < 0.05) was calculated according to the two-tailed exact Fisher test for contingency tables and using a Mann-Whitney rank sum test for the comparison between cases and controls in the case of continuous variables. To study the association between having had or not more than 5 previous ED visits for injuries and having or not having symptoms suggestive of ADHD, we conducted a bivariate (thus unadjusted) logistic regression analysis. All analyses were done using Stata/IC 14.2 (StataCorp LLC, College Station, USA).

## Results

**Fig. 1:**
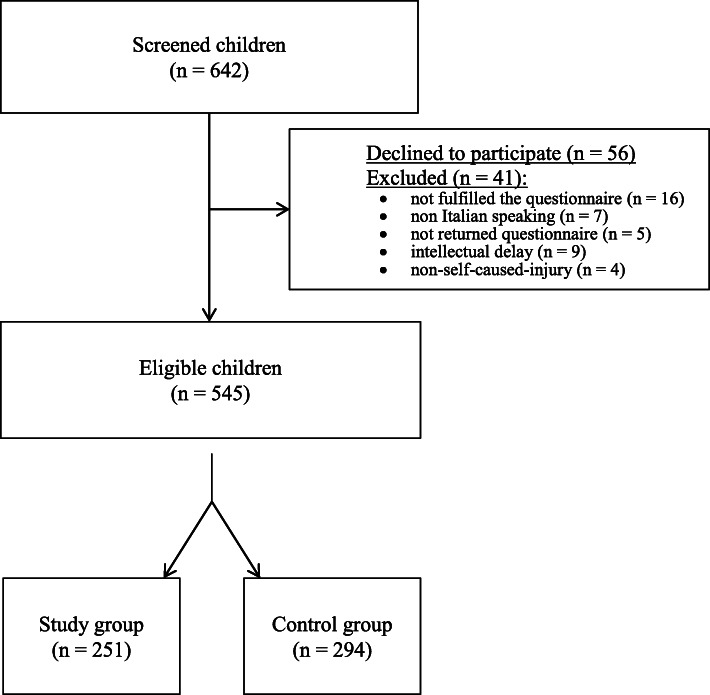
Flow-chart of the study

During the study period, 642 eligible children were approached. Fifty-six of them declined to participate and 41 patients were excluded from the study (16 did not complete the questionnaire, seven were unable to communicate in Italian, five left the ED without returning the questionnaire, nine presented intellectual delay, four had non-self-caused-injuries) (Fig. 1). We enrolled 545 children aged between 6 and 17 years. Among these, 251 patients visited the ED for injuries (46%), and 294 visited for other causes (54%).

The characteristics of the enrolled patients are described in Table 1. Cases and controls did not differ significantly regarding age. However, the number of males was significantly higher in the case group. The 294 patients enrolled in the control group attended our ED for the following reasons: 176 for infections, 31 for gastrointestinal diseases excluding infection, 27 for dermatological problems, 24 for neurological diseases, 14 for cardiological diseases, 11 patients for orthopaedic reasons other than trauma, eight for gynaecological diseases, and three for endocrinological and haematological diseases.

**Table 1:** Description of cases (patients attending the emergency department for injuries) and controls (patients attending for other causes but injuries). Numbers in the table are frequencies and percentages or medians and interquartile ranges. *P*-values are the result of two-tailed Fisher exact tests for categorical variables and Mann-Whitney rank-sum tests for continuous variables

	**Cases (251)**	**Controls (294)**	***p-*value**
**Sex**	Males = 164 (65%)	Males = 139 (47%)	*p* = 0.000
**Age (years)**	11.4 (8.6–13.8)	10.6 (8.1–14.3)	*p* = 0.424
**Triage code**	White, 46 (18%) Green, 187 (75%) Yellow, 18 (7%)	White, 97 (33%) Green, 175 (60%) Yellow, 22 (7%)	*p* = 0.000
**For cases: type of injury For controls: main diagnoses**	Trauma, 143 (57%) Minor wound, 29 (12%) Major wound, 26 (10%) Compound fracture, 42 (17%) Displaced fracture, 11 (4%)	Infection, 176 (60%) Gastrointestinal excluding infection, 31 (10%) Dermatologic, 27 (9%) Neurologic, 24 (8%) Cardiologic, 14 (5%) Ortopedics excluding trauma, 11 (4%) Ginecologic, 8 (3%) Endocrinologic/hematologic, 3 (1%)	

Among all patients, 37 children (7%) obtained a score higher than or equal to 14 in the questions related to inattention, and 28 children (5%) obtained a score higher than or equal to 12 in the hyperactivity/impulsivity questions.

Among the children who participated in our ED due to injury, 22 showed behaviours suggestive of ADHD, while in the control group, there were 30 without statistically significant differences between the groups (*p* = 0.661) (Table 2).

**Table 2:** Differences between cases and controls for SCOD scores and ADHD related items. Numbers in the table are frequencies and percentages or median and interquartile ranges. *P-*values are the result of two-tailed Fisher exact tests for categorical variables and Mann-Whitney rank-sum tests for continuous variables

	**Cases (251)**	**Controls (294)**	***P-*value**
**Previous ADHD diagnosis**	10 (4%)	4 (1%)	*p* = 0.061
**Inattention score (items 1 to 9)**	4 (1–8)	4 (1–7)	*p* = 0.395
**Inattention score ≥ 14**	15 (6%)	22 (7%)	*p* = 0.501
**Impulsivity/hyperactivity score (items 10 to 18)**	2 (1–5)	3 (0–5)	*p* = 0.859
**Impulsivity/hyperactivity score ≥ 12**	13 (5%)	15 (5%)	*p* = 1.000
**ADHD suggestive symptoms (positivity to SCOD-G questionnaire)**	22 (9%)	30 (10%)	*p* = 0.661

Children with scores evocative of ADHD (*n* = 22) showed a probability four and a half times higher (OR 4.52; 95% CI 1.45–14.04; *P* = 0.009) of having had more than 5 previous ED visits for injuries, compared to the children of the same group without ADHD behaviours (*n* = 229). On the other hand, there was no difference in the frequency of past fractures, the need for previous suturing or antecedent injury-related hospitalisations in children with scores suggestive of ADHD (Table 3).

**Table 3:** Patients attending the emergency department for injuries (cases): differences between patients with positivity vs. negativity to ADHD suggestive symptoms. *P-*values are the result of two-tailed Fisher exact tests for categorical variables and Mann-Whitney rank-sum tests for continuous variables

	P-SCOD (22)	N-SCOD (229)	TOTAL (251)	*p-*value
***Sex***				***P = 1.000***
Females	7 (32%)	80 (35%)	87 (35%)	
Males	15 (68%)	149 (65%)	164 (65%)	
***Age***	10.3 (8.3–13.0)	11.5 (8.7–13.9)	11.4 (8.6–13.8)	***P = 0.426***
***Triage code***				***P = 0.389***
white	4 (18%)	42 (18%)	46 (18%)	
green	15 (68%)	172 (75%)	187 (75%)	
yellow	3 (14%)	15 (7%)	18 (7%)	
red	0	0	0	
***N. of previous ED accesses***				***P = 0.017***
≤ 5	17 (77%)	215 (94%)	232 (92%)	
> 5	5 (23%)	14 (6%)	19 (8%)	
***Previous sutures***				***P = 0.268***
*None*	10 (45%)	133 (58%)	143 (57%)	
*1–5*	12 (55%)	96 (42%)	108 (43%)	
***Previous fractures***				***P = 0.230***
*None*	18 (82%)	155 (68%)	173 (70%)	
*1–9*	4 (18%)	73 (32%)	77 (30%)	
***Previous hospitalizations related to injuries***				***P = 0.071***
*none*	17 (77%)	207 (90%)	214 (85%)	
*1–5*	5 (23%)	22 (10%)	27 (10%)	

## Discussion

This study did not show any differences in behaviours suggestive of ADHD in children and adolescents visiting the ED for injuries compared to other causes. Nevertheless, we detected a population of patients with behaviours suggestive of ADHD whose history was remarkable for numerous injury-related ED visits.

Previously published evidence is discordant about the effects of ADHD on children attending the ED because of trauma: one study showed a 3-fold higher risk of being affected by ADHD than controls. Another similarly found a double risk of having higher scores on the Conners’ scale [8, 19]. However, these results were challenged by another report, which showed that children visiting the ED with injuries were no more likely than non-injured children to have unrecognised ADHD, based on parental screening [9].

It is not possible to diagnose ADHD within the ED setting, and nor was this the aim of this study. Nevertheless, this report shows that the core behaviours of ADHD, such as inattention and hyperactivity/impulsivity, could be explored with a brief and focused questionnaire. Surprisingly, we found a population of children with behaviours suggestive of ADHD who had numerous previous injury-related ED visits. This finding supports previous studies that show that a history of repeated traumas, head injuries, or burns are potential indicators of ADHD [20, 21].

Injuries are the most common complication due to the hyperactivity and inattention, and children with ADHD have double the risk of dying compared to peers without ADHD. This increased risk is related to accidents, and it rises to five times by adulthood [14]. Specific treatment for ADHD reduces comorbidities such as injury rate and trauma-related ED visits by up to 43%, as well as substance use disorders, behavioural impairments and severe traffic accidents [22]. The authors underline that specific therapy appears to be more effective the sooner it is started [23]. With specific treatment, we are able to reduce the risk of injury and death; therefore, we should consider the implementation of effective screening strategies to detect ADHD in the ED. Future studies should investigate the efficacy of a questionnaire used in the ED for patients with a history of repeated injuries, to maximise the chances of early detection of children with behaviours suggestive of ADHD. These measures may allow early diagnosis, preventing severe ADHD-related impairments, which could occur from childhood to adulthood.

This study presents some limitations. First of all, the limited sample size and the lack of red codes may have conditioned the results of the primary outcome. We decided to exclude children with developmental delay, as a category of patients at higher risk for symptoms of ADHD. Moreover, we were not able to enrol patients 24 h a day, so it can be a possible bias. We also took into consideration only one part of the SCOD questionnaire, intending to focus only on behaviours considered highly suggestive of ADHD. Moreover, our cut-off scores suggestive of ADHD were based on symptoms in males, thus only including females with high scores. We did not perform a separate analysis for already diagnosed ADHD patients in which the treatment may have influenced the symptoms’ scores. Finally, we used rating scales based exclusively on subjective observations of parents and we therefore cannot exclude some recall bias regarding the secondary outcomes.

## Conclusion

In this series, the behaviours suggestive of ADHD were not presented more frequently in patients who visited the ED for lesions compared to other causes, so our results do not support a screening for ADHD symptoms in the ED. Nevertheless, we found a population of patients with behaviours suggestive of ADHD and an unusual history of injury-related ED visits. This specific population of children may benefit from a tool to identify ADHD suggestive symptoms in the ED. Future studies could be aimed at developing and validating a specific questionnaire and better identifying children who could benefit from this screening.

## Data Availability

The datasets used and analyzed during the current study are not publicly available due to their containing information that could compromise the privacy of study participants, but are available from the corresponding author on reasonable request.

## Abbreviations

ADHD: Attention-deficit/hyperactivity disorder
ED: Emergency department
IQR: Interquartile ranges
SCOD: Scala di valutazione dei Comportamenti Dirompenti, i.e. Evaluation Scale of Disruptive Behaviour
